# Biopsychosocial barriers affecting recovery after a minor transport‐related injury: A qualitative study from Victoria

**DOI:** 10.1111/hex.12907

**Published:** 2019-06-03

**Authors:** Stella Samoborec, Darshini Ayton, Rasa Ruseckaite, Susan M Evans

**Affiliations:** ^1^ Department of Epidemiology and Preventive Medicine (DEPM), School of Public Health and Preventive Medicine Monash University Melbourne Victoria Australia

**Keywords:** compensation, injuries, recovery, rehabilitation, transport accidents, trauma

## Abstract

**Objective:**

The aim of the study was to understand the recovery phenomena and to explore participants' perspectives on the biopsychosocial facilitators and barriers affecting their recovery after a minor transport injury.

**Methods:**

A qualitative method was used involving semi‐structured interviews with 23 participants who sustained a minor transport injury. Interviews and analysis were guided by the biopsychosocial model (BPS) of health. The outcomes were themes capturing biopsychosocial barriers to, and personal experiences of, recovery using a previously defined framework.

**Results:**

The themes indicate that recovery is a multifaceted phenomenon affected by comorbidities such as chronic pain, depression and anxiety. A range of subsequent complexities such as the inability to self‐care and undertaking daily domestic duties, and incapacity to participate in recreational activities were major barriers to recovery. These barriers were found to be an on‐going source of frustration, dissatisfaction and a perceived cause of depressive symptomatology in many participants. Most participants reported mixed feelings of the care received. Other common issues raised included a lack of understanding of the assessment time, regular follow‐up, guidance and on‐going support.

**Conclusion:**

This study revealed that recovery after a minor transport‐related injury was a challenging, complex, demanding and a long‐term process for the individuals in this study. Findings from this limited cohort suggested that, for participants to return to their pre‐accident health status, a more coordinated approach to information and care delivery may be required.

## 1 **INTRODUCTION**

Road trauma is a leading cause of disability and mortality in the world, and it results in more disability‐adjusted life‐years than any other chronic disease.^1^, ^2^ Worldwide, road transport accidents contribute substantially to the number of deaths and to the burden of disability.^3^, ^4^ The World Health Organization (WHO) estimates that by 2020 road accidents will be the third leading cause of disability. Most reported transport accidents result in minor injuries (75%), mostly musculoskeletal and/or soft tissue injuries.^5^ The injury itself, regardless of severity, may lead to permanent mental and physical impairments and disabilities.^6^, ^7^, ^8^ Even though minor injuries comprise approximately 75% of all road transport injuries, poor physical and mental health outcomes in this group have received little attention.^9^ Among participants with minor injuries, there are individuals at high risk of substantial poor recovery and on‐going disability.^7^, ^10^ Hence, permanent or temporary disabilities arising from minor injuries pose an on‐going problem for the compensation and health‐care system.^11^, ^12^ It has been acknowledged that this particular group is at risk of being left alone with problems and symptoms that are difficult to understand and often invisible to others.^13^, ^14^

Previous research indicates differences in factors affecting recovery, yet the most commonly reported factors are high pain intensity, chronic pain, older age, pain catastrophizing, poor recovery expectations and poor pre‐accident health status.^15^ Other reasons suggested for a poor recovery, or even for permanent disability, include factors other than purely injury‐related ones. Few qualitative studies have identified factors that largely depend on a person's environment and health‐care and compensation systems.^13^, ^16^ Others reported factors such as lack of family support,^17^ legal involvement^18^, ^19^ and the compensation claim.^20^, ^21^ However, it is still unclear how these factors impact the recovery outcome; how they interact; and which from an injured person's perspective are the most important to measure to identify people at high risk for poor recovery. There is currently no standardized definition of what poor recovery means, yet in this study, it was defined as chronic pain, depression, anxiety, incapacity to return to work, limited physical activity and mobility, limitations in daily living and similar symptomatology that prevents patients to return to their pre‐accident health state.

The academic literature posits that disability and recovery is best understood and managed using the BPS model, which systematically considers biological, psychological and social factors, as well as their complex interaction in understanding health and disability.^22^ The International Classification of Functioning, Disability and Health framework (ICF) provides a multi‐perspective, biopsychosocial approach to describing and measuring disability^23^ and has been used by other studies to understand factors impacting health outcomes road trauma.^24^, ^25^, ^26^, ^27^ Recent work has extended the BPS to consider its application in a transport compensation setting.^28^

Victoria is a state in south‐eastern Australia and has a population of 5.4 million, representing 25% of the national census.^29^ The Transport Accident Commission (TAC) is a Victorian Government‐owned organization set up to pay for treatment and benefits for people injured in transport accidents, promote road safety and improve Victoria's road trauma system.^26^ The TAC recognized the importance of better understanding minor injury claims and individual needs of the claimants, mainly because of the high cost involved in managing their claims, but also because approximately 20% of those who claim do not recover even 7 years after their accident.^30^ As such, there is an urgent need to further explore reasons for poor recovery to better understand minor injury claims and individual needs of the claimants and identify those at high risk of poor recovery.

The primary aim of this research study was to understand, from injured persons' perspective, the biological, psychological and social factors impacting their recovery outcomes; the recovery processes; quality of care provided to participants; and common social impacts. By understanding these issues, we anticipate earlier identification of people at risk of poor recovery, thereby enabling the development of intervention strategies to address barriers and accelerate recovery.

## 2 MATERIALS AND METHODS

### 2.1 Study setting

Participants in this study had sustained a minor transport‐related injury and lodged a compensation claim at the TAC. Injuries classified as minor, and involved in this study, included sprain, strain, whiplash‐associated disorder, contusion, abrasion, laceration or subluxation and any clinically associated sequelae, as defined by Minor Injury Guidelines.^31^

### 2.2 Sampling strategy

The qualitative study sample was drawn from participants who contributed to the TAC's annual Client Outcome Study (COS) survey (financial year 2015/16). The survey is of cross‐sectional design, had commenced in 2009 and annually collects clinical and health outcome data on a randomly selected group of claimants with non‐catastrophic injuries. The 2015/16 survey was completed via telephone with 1105 people aged between 16 and 89 years who had claim duration of at least 5 months.

Patient‐reported outcome measures (PROMs) collected by the COS include a single‐item recovery measure called Life Back on Track (LBoT) and the health‐related quality of life measure EQ‐5D‐3L. The LBoT is a TAC's internal overarching measure of recovery that captures participants' perspectives of how they feel about getting their life back on track after the accident. The LBoT scale ranges from 1 (ie life not back on track or poor recovery) to 10 (ie life back on track or good recovery) (TAC's internal source). The EQ‐5D‐3L is a standardized health utility instrument, and its summary index scores were used to describe health outcomes for participants involved in this study. It ranges from −0.594 to 1 with a score of <0.35 representing poor health, scores between 0.35 and 0.79 representing moderate health and scores between 0.80 and 1.00 representing good health.^32^

### 2.3 Profile of participants in the COS survey

The mean age of the 2015/16 survey participants was 48 years of age; there were more males than females (56% and 45%, respectively); and the majority were English speaking (91%) and were considered as participants for whom a certain level of recovery was expected based on their initial injury status (94%). Additionally, most participants were employed at the time of the survey (72%) and almost half had suffered minor injuries (49%).

### 2.4 Inclusion criteria

Participants were eligible to be included in this qualitative research study if during the COS survey they expressed an interest in participating in future research. This study was aimed at exploring the differences between recovered and non‐recovered individuals, by discussing their reported barriers and facilitators of recovery. The LBoT, a single‐item recovery instrument, was used to divide the participants into a ‘good recovery' group and a ‘poor recovery' group. A cut‐off score of 7 on the LBoT has routinely been used by the TAC to distinguish recovered (LBoT score ≥7) from non‐recovered (LBoT score <7) participants and was used in this study as well.

### 2.5 Qualitative study design

This qualitative study aimed to understand a wide range of experiences of, and barriers to, recovery of traffic accident victims who sustained a minor transport‐related injury. The study anticipated gaining an understanding of recovery, particularly focusing on aspects which were most important to participants. This approach allowed each participant's voice to be ‘heard' and to clearly discuss what influenced their experience of recovery. With this view, we sought to establish a more holistic view of recovery, which was person‐centred and focused on multiple layers of recovery (following a previously developed framework) and their complex interaction.

### 2.6 Conceptual framework and interview guide

The interview guide (Table 1) was developed in a way to allow the exploration of each topic across the biological, psychological and social domains of the framework.^28^ Some interview topics aimed to explore one domain, while others were expected to explore two or three pertinent domains. For example, the first topic ‘general health and the role of general practitioners' explored both the biological (medical history and health status) and the social domain (the health‐care system and culture), whereas ‘expectations of recovery after injury' explored only the psychological domain of the framework. Apart from this, each topic was designed to explore both the factors and outcomes within the framework. Additionally, multifaceted interaction of these factors, within each domain, was explored in detail.

**Table 1:** Interview guide developed for the purposes of this study based on the previously defined biopsychosocial conceptualized framework

Interview guide and key topics for exploration
1. Pre‐accident physical and mental health
2. General health and the role of general practitioners
3. Mental health and the role of mental health specialist
4. Personal needs and expectations of recovery after injury
5. Family support
6. Emotional state and coping skills
7. Quality of health care and access to relevant medical services
8. Social and community support
9. Return to work and/or independence and/or usual activities
10. Compensation process and quality of the support and assistance provided

The interview guide and questionnaire were developed based on the current literature^23^, ^24^, ^33^, ^34^ by the main researcher and a qualitative research expert. The questionnaire contained a mix of direct and semi‐structured questions, which, during interviews, were expanded to capture individual experiences (supplementary material). This approach allowed participants to speak freely, especially about negative experiences or behaviours.

### 2.7 Recruitment and ethical considerations

The recruitment process was coordinated by the Compensation research manager. As per the study protocol, participants were sent an explanatory statement and an invitation letter via post or email explaining the purpose of the research, its benefits and relevant participation information. An opt‐out approach was used whereby participants were given 2 weeks to decline participation in the study. It was chosen because the inclusion criteria included only participants who had already denoted that they were happy to participate in future research activities. This approach was more likely to achieve a higher response rate compared to an opt‐in consent model. For people who did not actively decline participation, a researcher contacted them after the 2 weeks opt‐out period expired to arrange a time and place for the interview. Permission to record the interview was sought. The study was approved by the Monash University Human Research Ethics Committee (MUHREC 2016 0971 7666).

When contacted by telephone to arrange the interview, the participants were informed that interview questions will explore their experiences of recovery from transport‐related injury in depth. In addition, they could decline to respond to questions if they wished, and could end the interview at any stage. Participants were advised that their anonymity would be assured. For this study, it was very important to ensure that participants felt they could talk freely and in confidence about their experiences.

Recruitment was conducted in three phases to avoid recruiting more participants than required to gain data saturation. Data saturation defines the point at which no new themes are identified, and it is suggested that it is usually reached at around 12 interviews.^35^ This phased approach also enabled the researcher to review the interview questions at the conclusion of the first phase, and to allow adjustments to be made in subsequent interviews. The first phase was conducted between March and May 2017. Ten participants were interviewed during phase 1. After phase 1, purposive sampling was employed to ensure adequate representation of male participants and participants from regional areas. The second phase was conducted between May and August 2017. Ten participants were interviewed during phase 2. The final phase was conducted between August and September 2017, during which three participants were interviewed. The average time of the interview was approximately 1 hour.

Each interview was audiotaped and transcribed by the main researcher. To ensure rigour in data analysis, a second qualitative researcher blindly coded the data and developed concepts were reviewed and examined.^36^ There were no major discrepancies in coding between the two researchers.

### 2.8 Analysis

Audio files were transcribed verbatim and reflexive notes taken by the researcher. The process of data collection has been described as involving ‘spiral patterns of activity' where data were collected and analysed, feeding into the focus of further data collection.^37^

Repeated reading of transcripts was carried out after each interview to highlight and understand meaningful and dominant concepts. These were labelled in the first initial coding stage. As initial codes emerged, interview focus changed to understand the emerging concepts in more depth. Thus, following interviews were depended on themes and concepts highlighted in previous interviews.

Following final stages of the coding, meaningful categories and refined codes were created and described, using the aforementioned framework. The data were coded in NVivo, a qualitative research software. For comprehensive reporting throughout the article, we have used the COREQ guidelines^38^ (supplementary material).

## 3 RESULTS

The characteristics of the sample extracted from the COS survey can be seen in Table 2. These included age, gender, type of injury, region, marital status, LBoT score (TAC internal source), health‐related quality of life assessed with the EQ‐5D‐3L instrument^32^ and self‐reported levels of pain.

**Table 2:** Characteristics of the study participants

Characteristics of the study participants	Poor recovery LBoT <7 N (11)	Good recovery LBoT ≥7 N (12)
Age groups		
27‐40	0	3
41‐55	8	6
56‐70	2	2
70+	1	1
Gender		
Male	4	4
Female	7	8
Injury type		
Musculoskeletal/soft tissue	9	6
Other minor (contusions)	2	6
Region		
Metropolitan	7	11
Regional	4	1
Marital status		
Married	4	8
Never married	1	1
Divorced/separated	6	2
Widowed	0	1
LBoT score		
1‐6 (Not back on track or ‘poor recovery')	11	‐
7‐10 (Back on track or ‘good recovery')	‐	12
EQ‐5D‐3L		
Good health (0.80‐1. 00)	0	2
Moderate health (0.35 < 0.80)	5	7
Poor health (<0.35)	6	3
Self‐reported levels of pain		
Mild pain	1	6
Moderate pain	4	5
Severe pain	6	1

Data source: COS survey.

Six participants were admitted to hospital as a result of their accident; 10 were treated and discharged directly from the Emergency Department (ED) for follow‐up with their General Practitioner (GP); and seven were treated by their GPs without admission to hospital or ED. Participants in the ‘poor recovery' group (n = 11) were mostly aged between 41 and 55 years of age, females, divorced or separated, with self‐reported mild to moderate levels of pain and poor health‐related quality of life scores (EQ‐5D‐3L summary scores of <0.35). Participants in the ‘good recovery' group consisted mostly of participants aged between 41 and 55 years of age, females, living in the metropolitan area, who reported moderate health‐related quality of life scores (EQ‐5D‐3L summary scores of 0.35‐0.70) and mild to moderate levels of pain.

In this section, themes relating to the biopsychosocial recovery following minor injury are discussed. Due to the sampling issues, which heavily relied on a newly developed LBoT instrument, we were not able to report on facilitators to recovery, as most participants in the ‘good recovery' group reported not being in good recovery at the time of the interview. Therefore, they have focused their stories on barriers that impacted their recovery.

Table 3 outlines main themes emerging from the analysis with relevant subthemes. The quotes used to illustrate the themes are drawn from a range of participants from different age groups, time since accident and residential areas.

**Table 3:** Themes with relevant subthemes and additional quotes

Theme	Subtheme	Additional quotes
Biological Chronic pain, functional limitations and physical disability	1. Chronic pain and pain management	Well I'm in pain every single day. Someday, the pain is worse than others. Someday, I can't get out of bed. (Male, 58, Soft tissue – whiplash)
	2. Limitations to mobility and activities of daily living	I can't move, bend down, I can't lift anything anymore, it impacts on my shopping, I can't do big shopping at the time. (Female, 44, Soft tissue‐lover back)
	3. Inability to take part in former social and recreational activities	I can't run anymore, my leg won't allow me to run as I have problems with my knee now. (Male, 41, Leg laceration)
	4. Inability to return to work accompanied by financial hardship	I can't have my normal life. I have reduced my job dramatically. I'm now at the stage where I work 37 hours fortnightly and that's the maximum I can cope with. (Female, 44, Soft tissue‐lover back)
Psychological Mental health and psychological response to the traumatic event	5. Poor expectations of full recovery	My recovery expectations changed. The pain makes it harder, I feel down a lot of times. (Female, 44, Soft tissue‐whiplash)
	6. Anger and frustration due to unexpected recovery trajectory resulting in poor coping abilities	What really makes me angry that this woman, as she admitted that she was on her mobile and she was checking her schedule, and what makes me angrier it's that she got away with it and look at me, I will suffer for the rest of my life. (Female, 48, Soft‐tissue‐lower back)
	7. Anxiety and depression ‐ common comorbidities resulting from injuries	Yes obviously I'm depressed because I can't do much, I'm limited. (Female, 48, Soft tissue‐ whiplash)
Social Perceptions of poor quality of care and disappointments with the health system	8. Perceptions that assessments are not thorough which resulted in a ‘doctor shopping' behaviour and poor continuity in care	I had three GPs. The first one was terrible, totally ineffective, she didn't even do an assessment or send me for X‐ ray after I said I had a car accident and I'm in pain and that's why I went to see another one and another one. They were just incapable of doing their job. (Female, 45, Soft tissue‐ neck and shoulder)
	9. Perceptions of poor quality of care due to reluctance to deal with compensation clients and consequent lack of trust	It is very short assessment, very short follow up. Very basic, like they don't care or don't trust us, maybe because I was going through compensation. (Male, 36, Soft tissue‐ lover back)

### 3.1 Biological theme: Chronic pain, functional limitations and physical disability

#### 3.1.1 Subtheme: Chronic pain and pain management

This study identified that chronic pain was common among participants, regardless of their age or time since the accident. Chronic muscle pain and dysfunction were most commonly reported by participants who had sustained soft tissue injuries, such as whiplash and back injuries (sprains and strains).

Look I'm in pain, it's constantly with me since the car accident. It never goes away. When I say that I'm in pain that means that it reached 9‐10 [on a pain scale]. (Female, 44, Soft tissue‐ lower back)

Participants expressed there was lack of pain management provided by their health professionals or an obliviousness of the impact that pain was having on them. Eight participants described major dissatisfaction with pain management due to the increased consumption of analgesics, which caused severe side‐effects identified by the participants (eg impaired concentration, memory problems and incapacity to cope). Participants perceived that their chronic pain was poorly managed as health practitioners' attention was focused on treating symptoms rather than the underlying cause of the pain.

Well… I don't know. My doctor just fills me with pills and Lyrica and he just “masked” it and put it away. Lyrica is horrible I hate it. (Female, 48, Soft tissue‐neck and back)

#### 3.1.2 Subtheme: Limitations to mobility and activities of daily living

One of the major issues raised by participants was the inability to ambulate due to pain and the consequent disability it created. Most participants (n = 15) reported some degree of functional limitations that affected their daily life. This was particularly raised by younger and middle‐aged participants who used to be physically active before the accident. Participants reported with anger and frustration their inability to attend to simple tasks such as dressing and shopping. While some participants (n = 5) received home assistance support immediately following the accident, most (n = 14) did not receive any assistance with their daily activities.

Yes I was and I was able to walk and run but my health has changed in a lot of ways and basically it was chronic pain that was number one consequence of my accident because I can't do simple daily things as I used to. (Female, 50, Soft tissue‐ neck and back)

#### 3.1.3 Subtheme: Inability to take part in former social and recreational activities

Most participants discussed how involvement in sport or physical recreation offered many benefits, ranging from simple enjoyment to improved health and the opportunity for social interaction. Participation in sport was particularly important for participants who said that they were fit and physically active before their accident. However, due to the injury and current comorbidities (predominantly pain), many participants commented on not being able to return to pre‐accident recreational activities such as dancing and walking. They reported that not being able to participate in those activities negatively affected their social life and consequently their quality of life. This appeared to be independent of their age and type of recreational activity in which they were involved.

My health changed heaps, I used to exercise a lot, now I can't do it for more than 20 minutes. So, I don't do much. Everything I was doing basically I can't do anymore in a same way, which is frustrating and depressing. I will never get better. (Male, 62, Soft tissue‐contusion and whiplash)

#### 3.1.4 Subtheme: Inability to return to work accompanied by financial hardship

Several participants (n = 10) reported being unable to return to work due to injuries or comorbidities arising from their accident. Many (n = 9) reported that while physical injuries were more obvious and evident obstacles to returning to work, mental health issues were less recognized and accepted as valid reasons for not returning to work. Participants expressed frustration and disappointment with their current working arrangement as most had to reduce their working hours or reallocate and adjust their working positions. Some participants reported not being able to return to work at all which impacted their mental health state, for example by being less able to cope with symptoms of their injury and by lowering their expectations of ever reaching full recovery. This had also resulted in financial hardship, emotional stress and feeling of injustice to many participants.

I was not physically ready to go back … because when I was back to work like … I felt dizzy and not feeling well, I was walking around hanging on to the walls and I knew I shouldn't have gone back to work. (Female, 39, Soft tissue‐contusion)

### 3.2 Psychological theme: Mental health and psychological response to the traumatic event

#### 3.2.1 Subtheme: Poor expectations of full recovery

Recovery expectations were generally described as poor by participants. Despite some participants (n = 5) feeling they were initially positive about their hopes of a full recovery, many were not able to reach full recovery. Full recovery was perceived by a few participants as ‘getting back to where they were prior their accidents'. Many participants (n = 18) felt they would never fully or successfully recover or that they had reached the maximum extent of their recovery, which, in their case, was not full recovery. This was concerning to some participants, especially if they did not expect it. They also talked about feeling helpless in their recovery as they could not deal with chronic pain and side‐effects caused by medications they were taking. As chronic pain persisted for many years, participants expressed that they would never recover and had lost faith in achieving a good recovery outcome.

Look I have accepted I will never get better, it's been 4 years now and it's not improving. I tried everything possible and now have accepted that I will never be 100%. (Female, 44, Soft tissue‐back)

#### 3.2.2 Subtheme: Anger and frustration due to unexpected recovery trajectory resulting in poor coping abilities

Anger and frustration were common emotions expressed by the participants who articulated that they were not at fault for their accident. These participants described feelings of injustice and unfairness, as they were not the cause or reason for on‐going pain and disability. Some participants discussed not being able to adjust to the new situation, which became a source of on‐going daily frustration and anger.

Yes it's a problem for me. I get angry and depressed at the same time but I try to keep on with living. I volunteer in the local community centre, helping people because I can't work. Pain is killing me and the worse thing is that this was not my fault. (Female, 63, contusion, soft‐tissue‐hip)

#### 3.2.3 Subtheme: Anxiety and depression – common comorbidities resulting from injuries

Some participants described being ‘depressed' and ‘down' after their accident, while others described being anxious or moody. Many stated (n = 15) they had difficulty coping with the chronic pain, which seemed to be an on‐going source of their negative feelings and emotions. Many participants (n = 13) mentioned the complexity of their recovery while speaking about depression and anxiety.

I felt very sad and depressed about it. I could hardly drive, I couldn't do any work at home so I was very stressed and upset. I had to rely on other people and we don't have kids, we are alone here and my husband is working as well so I had to wait for him to go for the doctor's appointment. (Female, 46, Soft‐tissue‐arm)

Twelve participants reported receiving treatment for either anxiety, post‐traumatic stress disorder or depression by a health professional. Some participants (n = 4) described how their feelings of anxiety and depression were compounded by financial stress associated with their inability to return to pre‐injury work status.

I'm actually… since car accident, I was really depressed, I was vomiting and just crying and feeling so sorry for myself because I can't work and I have no income. I'm now on antidepressants. Terrible. (Female, 39, Soft tissue‐contusion)

### 3.3 Social theme: Perceptions of poor quality of care and disappointment with the health system

#### 3.3.1 Subtheme: Perceptions that assessments are not thorough which resulted in a ‘doctor shopping' behaviour and poor continuity in care

Fourteen of the 23 participants described a perception of poor quality of care. Three participants who had a long‐standing relationship with their GP were more satisfied with the care received compared with those who did not have a GP who was familiar with their medical history.

I was lucky because I had my GP, I've known them since I was a kid and they are really good. They care about me and my wellbeing. (Female, 48, Soft‐tissue‐neck)

Nonetheless, most participants (n = 16) reported having seen multiple GPs and other health professionals and also felt they were forced to ‘shop for a doctor' who was willing to help them. Participants perceived that this resulted in poor continuity of care, communication breakdown and inadequate assessments. Many (n = 10) reported that this was largely the result of short appointments of only 10 minutes, which did not allow for detailed discussion of their physical and mental health state. Some participants also reported the inability to build a rapport and bond with their health professionals, which resulted lack of trust and communication breakdown.

It was not thorough as it should be. I think she was just new and maybe cautious or overcautious and bit worried about TAC, doing claims with them …maybe. I have seen 5‐6 GPs and no one seemed to care or help. (Female, 48, Soft tissue‐ whiplash)

#### 3.3.2 Subtheme: Perceptions of poor quality of care due to reluctance to deal with compensation clients and participant's consequent lack of trust in clinical management

Participants (n = 17) raised concern about health professionals not eager to participate in their recovery process because they were going through compensation process, and that they did not trust in the persistence of their symptoms such as chronic pain and functional disability.

I'll be like trying to find a good GP is very difficult in itself. And even you know, I really didn't have a good GP. I tried to find someone who you can trust and talk to, not someone rejecting you because you claim compensation. (Female, 27, Soft tissue‐ whiplash)

## 4 DISCUSSION

To our knowledge, this is the first study in Victoria providing in‐depth information of participants' experiences of recovery after a minor transport‐related injury. Others have reported the recovery journey from the perspective of people who have sustained more serious injuries. We found that the complexities and challenges identified in this cohort were similar to those reported in participants who sustained more severe injuries.^39^, ^40^ As seen from the results, participants identified a number of barriers which affected their recovery.

A number of participants reported challenges with chronic symptoms, sometimes persisting for years. This was particularly evident in participants suffering chronic back pain and whiplash‐associated disorder. It is well‐known that high level of pain is common following injury and is a strong predictor of chronic pain.^41^, ^42^ In this cohort, the impact of injury was mostly related to unresolved limitation to physical function and chronic pain, which hampered participants' ability to participate in social, leisure and other activities of daily life. There was a perception among participants that poor pain management involving long‐term usage of pain medications seemed to have done more harm than good. Consistent with our study, others have reported similar findings in minor^16^ and severe^40^ injury cohorts.

Even though physical recovery seemed to be first priority during recovery, it was only one element of the overall recovery progress. Participants emphasized that the psychological and social components of their recovery were vital parts of their journey. As confirmed in previous studies, the complexity and duration of emotional impacts varies in trauma cohorts,^43^, ^44^, ^45^ as do the physical consequences. In this particular group, these impacts were largely related to an inability to work and negative experiences with recovery processes and complex system procedures.

From a social perspective, information, guidance, coordination and on‐going support throughout the recovery appeared to be vital for participants, regardless of the time since accident. Many participants described substantial difficulties with finding the ‘right' health professional to treat them, while others were still in process of finding one. Participants' perception was that their recovery was impacted by poor communication. Subsequent lack of trust between participants and health professionals seems to have led to numerous communication issues. The need for improved and enhanced provision of information, guidance and communication has been previously emphasized,^46^ and it is also confirmed in our study. There would be benefit in developing information protocols and guidelines for participants with poor recovery outcomes to address delays in finding an appropriate health professional willing to manage their care. Participants also expressed a need for a central point of contact for coordination of care but were unable to articulate who should provide this service. Given that GPs are often the first and on‐going point of contact, they might be suitable for this role. However, this would require addressing numerous barriers to GPs treating compensable injuries.^12^ Barriers reported by GPs included time and financial burdens, in addition to the clinical complexities involved in compensable injury management.

Lack of GP engagement is concerning from an ethical and financial perspective, and it is well‐known that timely intervention and coordinated treatment is crucial for successful recovery.^47^, ^48^

In summary, using the biopsychosocial framework in this study enabled detection of important obstacles and barriers to recovery. If these problems had been identified during the recovery process, tailored interventions could have been developed and recovery outcomes may have been improved. The results of this study call for a regular assessment of barriers affecting recovery through a targeted survey or an existing instrument that has the capacity to assess these barriers and complexities. An important next step would be to develop an approach and formulate questions to be asked in a larger similar cohort to examine the prevalence of issues raised as barriers to recovery among a larger cohort. If these are indeed issues, then strategies will need to be developed to appropriately address them.

A consistent and reliable point of contact for treatment, follow‐up and information is necessary to improve and provide support to facilitate better recovery outcomes. Improved clarity in delivering information and timeliness of the delivery of required medical services would be beneficial to improve the level of care and support.

However, participants' perceptions and observations require further exploration with health professionals, as they play a crucial role in facilitating better recovery outcomes.^26^ This is an area requiring further research, as it is still unclear why this cohort perceived to be left alone in managing their recovery, considering the amount of problems they experienced.

The combination of qualitative findings and subsequent information obtained through quantitative research will assist policy leaders in developing a longer‐term roadmap to assist patients who have sustained a minor injury with a protracted recovery path.

This research provides valuable and rich information about recovery after a minor transport‐related injury. However, there are a number of important limitations worthy of mention. Firstly, this study is not intended to be representative. Qualitative research is hypothesis‐generating. As such, we are unable to determine the extent to which issues raised by respondents are common among all minor transport injured victims. Secondly, the sample was limited to those who sought compensation for their injuries. We cannot assume that issues raised by this group are similar to those who do not claim for compensation. Third, respondent bias is likely among this cohort; even though the participants were told that their participation in the interview would not affect their claim, some may have not been truthful about their experiences of recovery for fear of how this may impact their claim. Fourth, we are unable to gauge the extent to which the symptoms expressed by participants such as anxiety and depression pre‐dated the injury. Next, we did not obtain opinions of health professionals who treat these patients and may not have supported their views. This is an important future research step which may help to identify strategies to engage clinicians in treating people with compensable injuries. Finally, we were unable to report on facilitators or enablers of recovery due to the insensitivity of the LBoT instrument to identify those who reported good or successful recovery.

## **CONFLICT OF INTEREST**

Authors have no conflict of interest to report. The data that support the findings of this study are available on request from the corresponding author. The data are not publicly available due to privacy or ethical restrictions.
