# Supplementary material for: Biopsychosocial barriers affecting recovery after a minor transport‐related injury: A qualitative study from Victoria
Source: Health Expect. 2019 Jun 3;22(5):1003–12. doi: 10.1111/hex.12907 (PMC6803416; doi:10.1111/hex.12907)
Supplement: Supplementary file 1 [file HEX-22-1003-s001.pdf]

Supplementary material 1

COREQ (Consolidated criteria for Reporting Qualitative research) Checklist

Topic	Item No.	Guide Questions/Description	Description	Reported on Page No.
**Domain 1: Research team and reflexivity**				
Personal characteristics				
Interviewer/facilitator	1	Which author/s conducted the interview or focus group?	Primary author	Page 8
Credentials	2	What were the researcher's credentials? E.g. PhD, MD	PHD candidate<br>M BMed Sci Epi	Title page
Occupation	3	What was their occupation at the time of the study?	Epidemiologist/public health researcher	Title page
Gender	4	Was the researcher male or female?	Female	NS
Experience and training	5	What experience or training did the researcher have?	Previous qualitative studies conducted and support from the qualitative research expert.	NS
Relationship with participants				
Relationship established	6	Was a relationship established prior to study commencement?	Yes.	
Participant knowledge of the interviewer	7	What did the participants know about the researcher? e.g. personal goals, reasons for doing the research	They were told the rationale behind this research, for what purpose it is and who is the researcher interviewing them.	Page 7
Interviewer characteristics	8	What characteristics were reported about the inter viewer/facilitator? e.g. Bias, assumptions, reasons and interests in the research topic	NS	NA
**Domain 2: Study design**				
Theoretical framework				
Methodological orientation and Theory	9	What methodological orientation was stated to underpin the study? e.g. grounded theory, discourse analysis, ethnography, phenomenology, content analysis	Grounded theory	Page 5 and 6
Participant selection				

Sampling	10	How were participants selected? e.g. purposive, convenience, consecutive, snowball	Purposive sampling was applied.	Page 6 and 7
Method of approach	11	How were participants approached? e.g. face-to-face, telephone, mail, email	Mail and telephone.	Page 7 and 8
Sample size	12	How many participants were in the study?	Not that many, they were willing to participate. Many were not contactable though.	Page 7
Non-participation	13	How many people refused to participate or dropped out? Reasons?	A few, mainly due to the timing.	Page 7 and 8
Setting				
Setting of data collection	14	Where was the data collected? e.g. home, clinic, workplace	Half was collected face-to-face. Other were held over the phone as some participants preferred having over the phone interview due to travelling, place arrangements etc.	Page 7
Presence of nonparticipants	15	Was anyone else present besides the participants and researchers?	No. Due to the privacy and confidentially issues. Only on first interview, there was the student' supervisor.	NS
Description of sample	16	What are the important characteristics of the sample? e.g. demographic data, date	Can be seen in Table 1	Page 8
Data collection				
Interview guide	17	Were questions, prompts, guides provided by the authors? Was it pilot tested?	Supplementary material . Questionnaire was developed based on literature review and conceptualised framework. It was piloted during the first 5 interviews and adjusted accordingly. As it was grounded theory, we have focused on some problems and issues more in subsequent interviews.	Page 6 and 7
Repeat interviews	18	Were repeat inter views carried out? If yes, how many?	No. It was not needed.	NS
Audio/visual recording	19	Did the research use audio or visual recording to collect the data?	Audio was used.	Page 7 and 8
Field notes	20	Were field notes made during and/or after the inter view or focus group?	Yes noted were always made.	Page 7 and 8
Duration	21	What was the duration of the inter views or focus group?	Approximately 1 hour. Some were much longer than that.	Page 7

Data saturation	22	Was data saturation discussed?	Yes it was. It was reached before we finished, but due to the methodology we wanted to continue to be able to elaborate on theory behind recovery and what it constitutes and how important are these barriers in recovery processes and outcomes.	Page 7
Transcripts returned	23	Were transcripts returned to participants for comment and/or	No. We didn't see it needed. But the articles will be send to them once published.	NS
**Topic**	**Item No.**	**Guide Questions/Description**		**Reported on Page No.**
		correction?		
**Domain 3: analysis and findings**				
Data analysis				
Number of data coders	24	How many data coders coded the data?	Two.	Page 8
Description of the coding tree	25	Did authors provide a description of the coding tree?	Yes.	Page 8
Derivation of themes	26	Were themes identified in advance or derived from the data?	Derived from the data.	Page 6 and 8
Software	27	What software, if applicable, was used to manage the data?	NVivo	Page 8
Participant checking	28	Did participants provide feedback on the findings?	No, but the interviews were always summarised to them at the end based on what they said to make sure we understood them and interpreted them correctly.	NS
Reporting				
Quotations presented	29	Were participant quotations presented to illustrate the themes/findings? Was each quotation identified? e.g. participant number	Yes. We have used more additional quotes to make the findings more robust.	Page 9 to 14 Table 3
Data and findings consistent	30	Was there consistency between the data presented and the findings?	Yes. Only the consistent themes were reported.	Page 8
Clarity of major themes	31	Were major themes clearly presented in the findings?	Yes, definitely.	Table 3
Clarity of minor themes	32	Is there a description of diverse cases or discussion of minor themes?	Not in this article, as we produced 2 articles from this study. One was looking into general barriers reported by patients, and another (published elsewhere) just	NS

			focused on compensation system as this was a big theme in the study and many sub-themes were developed within that theme. This complex phenomena was therefore described in a separate study.	

Developed from: Tong A, Sainsbury P, Craig J. Consolidated criteria for reporting qualitative research (COREQ): a 32-item checklist for interviews and focus groups. *International Journal for Quality in Health Care*. 2007. Volume 19, Number 6: pp. 349 – 357

Supplementary material 2

Interview questions

I am going to begin our interview today with asking you some questions about yourself and your health.

1. How old are you?
2. What is your occupation?
3. What was the highest level of education you achieved?
4. Do you live with anyone? If yes, how many people and who are they?
5. Do you have a healthcare card/pension card/private health card?
6. When did your injury occur?
7. How much has your health changed since the accident?
8. Are you taking any medications related to your injury?
    - If yes, what type of medications and how frequently? Who has prescribed these medications?

I am now going to ask you about your accident and injury and how it has impacted your health?

9. Can you tell me about your accident?
    - Circumstances and injury details
10. Can you describe what impact the accident has had on your health and wellbeing?
    - What was your health status before the accident and after (exercise, mental wellbeing, and ability to do activities of daily living)?
11. Can you tell me about your recovery?
    - What do you mean by recovery? How do you know if you have successfully recovered?
    - If not recovered, what are your expectations for your recovery?

I am now going to ask you questions about the health professionals you may have seen during your recovery and the health care you have received.

12. What was the role of your general practitioner in this recovery process?
13. Could you describe to me your visits to your GP?
    - Information provided? How many of visits (approximately)? How many GP's?
14. Has an allied health professional (e.g. a physiotherapist) had a role in this recovery process?
    - Information provided? Number of visits? How many professionals?

15. What was the role of a mental health specialist in your recovery process?
16. Could you describe to me your visits to your mental health specialist?
    - What type of specialist? Information provided? Number of visits? How many specialist?
17. If you have been admitted to hospital, how satisfied were you with the health care received?
18. How satisfied were you with the quality of medical services and medical providers during your recovery?
    - If you were not satisfied, could you please explain to me why and what would you change? Do you think this has impacted your outcomes and how much?
19. During your recovery, did you have access to medical services and health care you needed? If not, can you please explain why?

I am now going to ask you questions about the support you may have received during your recovery and your personal experiences of recovery.

20. How would you describe support and help received from your family and friends?
21. How would you describe support and help received from your community?
22. Can you describe to me your expectations regarding return to work and/or usual activities?
    - Did expectations change during the recovery process?
23. How would you describe support and assistance you received from the TAC during your recovery?
    - Are you happy with the level of support provided by the TAC?
    - What could the TAC have done better?
    - Can you describe to me the type and process of your compensation claim?
    - Was a solicitor involved?
    - In your opinion, has the compensation process impacted and prolonged your recovery?
    - If yes, could you please explain to me why and what would you change?
    - In your opinion, what part of compensation process had the greater impact on your recovery?
24. In your opinion, what are the most important key factors for a person to recover after injury?
    - What was most important for you?
25. Where do you see yourself in 2 years' time?
    - To what extent have you been able to get your life back on track? (back to work and/or your usual daily activities)
26. Is there anything else you would like to share with me about your recovery and health?
