# Supplementary material for: Biopsychosocial barriers affecting recovery after a minor transport‐related injury: A qualitative study from Victoria
Source: Health Expect. 2019 Jun 3;22(5):1003–12. doi: 10.1111/hex.12907 (PMC6803416; doi:10.1111/hex.12907)
Supplement: Supplementary file 2 [file HEX-22-1003-s002.docx]

**​Supplementary material 2**

Interview questions

I am going to begin our interview today with asking you some questions about yourself and your health.

1. How old are you?
2. What is your occupation?
3. What was the highest level of education you achieved?
4. Do you live with anyone? If yes, how many people and who are they?
5. Do you have a healthcare card/pension card/private health card?
6. When did your injury occur?
7. How much has your health changed since the accident?
8. Are you taking any medications related to your injury?

- If yes, what type of medications and how frequently? Who has prescribed these medications?

I am now going to ask you about your accident and injury and how it has impacted your health?

1. Can you tell me about your accident?

- Circumstances and injury details

1. Can you describe what impact the accident has had on your health and wellbeing?

- What was your health status before the accident and after (exercise, mental wellbeing, and ability to do activities of daily living)?

1. Can you tell me about your recovery?

- What do you mean by recovery? How do you know if you have successfully recovered?
- If not recovered, what are your expectations for your recovery?

I am now going to ask you questions about the health professionals you may have seen during your recovery and the health care you have received.

1. What was the role of your general practitioner in this recovery process?
2. Could you describe to me your visits to your GP?

- Information provided? How many of visits (approximately)? How many GP’s?

1. Has an allied health professional (e.g. a physiotherapist) had a role in this recovery process?

- Information provided? Number of visits? How many professionals?

1. What was the role of a mental health specialist in your recovery process?
2. Could you describe to me your visits to your mental health specialist?

- What type of specialist? Information provided? Number of visits? How many specialist?

1. If you have been admitted to hospital, how satisfied were you with the health care received?
2. How satisfied were you with the quality of medical services and medical providers during your recovery?

- If you were not satisfied, could you please explain to me why and what would you change? Do you think this has impacted your outcomes and how much?

1. During your recovery, did you have access to medical services and health care you needed? If not, can you please explain why?

I am now going to ask you questions about the support you may have received during your recovery and your personal experiences of recovery.

1. How would you describe support and help received from your family and friends?
2. How would you describe support and help received from your community?
3. Can you describe to me your expectations regarding return to work and/or usual activities?

- Did expectations change during the recovery process?

1. How would you describe support and assistance you received from the TAC during your recovery?

- Are you happy with the level of support provided by the TAC?
- What could the TAC have done better?
- Can you describe to me the type and process of your compensation claim?
- Was a solicitor involved?
- In your opinion, has the compensation process impacted and prolonged your recovery?
- If yes, could you please explain to me why and what would you change?
- In your opinion, what part of compensation process had the greater impact on your recovery?

1. In your opinion, what are the most important key factors for a person to recover after injury?

- What was most important for you?

1. Where do you see yourself in 2 years’ time?

- To what extent have you been able to get your life back on track? (back to work and/or your usual daily activities)

1. Is there anything else you would like to share with me about your recovery and health?
